# Supplementary material for: Characterizing mobility patterns and malaria risk factors in semi-nomadic populations of Northern Kenya
Source: PLOS Glob Public Health. 2024 Mar 13;4(3):e0002750. doi: 10.1371/journal.pgph.0002750 (PMC10936864; doi:10.1371/journal.pgph.0002750)
Supplement: S1 Text — (DOCX) [file pgph.0002750.s002.docx]

**S1 Text. Using Reported departure and return dates instead of the enrollment and follow-up dates**

The majority of departure (50%, 29/58) and return (51.7%, 30/58) dates had survey administration dates and participant reported dates within one day of each other. Discrepancies of > 1 week were observed for 2 start dates and 3 return dates, and could be indicative of a number of reasons including: recall bias for dates, a change in plans from when the traveler had originally told the CHW they would be departing, or challenges with the CHW being alerted to or reaching the traveler upon their return. The main text took a conservative approach to ensuring all travel might be captured by using the dates of enrollment and follow-up to define the travel period. Here, the trip data were reassessed using the departure and return dates reported by participants.

Generally, the trip type categorizations were the same (**S1 Table**) with 5.2% (3/58) of logged trips being categorized differently (one Long Term trip became Static, and two Transient trips became Long Term). Overall, the general trends described in the main text hold true: the most common trip type was still Long Tterm, followed by Static, Transient, and Day trips (**S1 Table**). Trip details, such as number of campsites logged, distance traveled, animals traveled with, and proportion of travelers reporting non-household members near camps, and water sources were similar across trip types. Consequently, it was concluded that using the enrollment and follow-up date to define the travel window was acceptable.
