# Supplementary material for: Characterizing mobility patterns and malaria risk factors in semi-nomadic populations of Northern Kenya
Source: PLOS Glob Public Health. 2024 Mar 13;4(3):e0002750. doi: 10.1371/journal.pgph.0002750 (PMC10936864; doi:10.1371/journal.pgph.0002750)
Supplement: S2 Text — (DOCX) [file pgph.0002750.s003.docx]

**S2 Text. Sensitivity analysis around definition of “long term campsite”**

A sensitivity analysis was run to determine the impact of the number of consecutive nights used to define a “long term camp” on the subsequent trip categorization. Because “Day trips” and “Static” trip categories were defined by spending at least 90% of their nights at the same location and were not impacted by the definition of a long term campsite, the following analysis just considered the changes between Transient and Long term trip categories when a “long term camp” was defined as spending 2, 7, or 14 consecutive nights at a location. As the number of nights increased, the proportion of travelers categorized as taking transient trips increased from 9.1% (4/44) to 70.5% (31/44) (**S2 Table**). Some characteristics were not sensitive to the number of consecutive nights: the demographics of the travelers remained similar (more females went on long term than transient trips, the median age for both groups was upper 30s); the most common animal traveled with was goats followed by sheep and then camels; more travelers used open water sources, rather than closed; and the majority of people in either trip category reported non-household members near their camps. While the median number of campsites logged and distances traveled per trip type did change as a function of the long term camp definition, the trend that Long term trips logged fewer campsites and traveled shorter distances, relative to Transient trips was upheld. Of the GPS logger carriers who had PCR results from follow-up, the prevalence of PCR positive cases detected was highest in the Long Term trip group when long term camps were < 7 nights and then similar between Long term and Transient trip groups when camps were 14 nights.
