# Supplementary material for: Characterizing mobility patterns and malaria risk factors in semi-nomadic populations of Northern Kenya
Source: PLOS Glob Public Health. 2024 Mar 13;4(3):e0002750. doi: 10.1371/journal.pgph.0002750 (PMC10936864; doi:10.1371/journal.pgph.0002750)
Supplement: S1 Table — (DOCX) [file pgph.0002750.s004.docx]

**S1 Table.** Comparison of characteristics of trips defined by different travel windows.

|  | **Survey dates** | | | | **Reported dates** | | | |
| --- | --- | --- | --- | --- | --- | --- | --- | --- |
|  | **Long term:** | **Transient:** | **Daytrips:** | **Static:** | **Long term:** | **Transient:** | **Daytrips:** | **Static:** |
|  | **(N = 24)** | **(N = 20)** | **(N = 3)** | **(N = 11)** | **(N = 25)** | **(N = 18)** | **(N = 3)** | **(N = 12)** |
| **Traveler details** |  |  |  |  |  |  |  |  |
| Male | 87.5 (21/24) | 100 (20/20) | 100 (3/3) | 90.9 (10/11) | 92.0 (23/25) | 100 (18/18) | 100 (3/3) | 83.3 (10/12) |
| Age (years) | 37 (30.0 - 48.5) | 36 (28.8 - 36.0) | 26 (22-44) | 38 (33-46) | 37 (30-48) | 36(29-39) | 26 (22-44) | 37 (34-45) |
| Catchment area |  |  |  |  |  |  |  |  |
| Kangirisae | 29.2 (7/24) | 30 (6/20) | 0 (0/3) | 18.2 (2/11) | 28 (7/25) | 33.3 (6/18) | 0 (0/3) | 16.7 (2/12) |
| Lowae | 37.5 (9/24) | 5 (1/20 | 33.3 (1/3) | 36.4 (4/11) | 32 (8/25) | 5.6 (1/18) | 33.3 (1/3) | 41.7 (5/12) |
| Nakurio | 8.3 (2/24) | 35 (7/20) | 0 (0/0) | 36.4 (4/11) | 12 (3/25) | 33.3 (6/18) | 0 (0/3) | 33.3 (4/12) |
| Kerio | 25 (6/24) | 30 (6/20) | 66.7 (2/3) | 9.1 (1/11) | 28 (7/25) | 27.8 (5/18) | 66.7 (2/3) | 8.3 (1/12) |
| **Trip details** |  |  |  |  |  |  |  |  |
| Trip duration (days) | 56 (41.5-77.5) | 55 (33.8 - 83.8) | 53 (41-92.5) | 70.0 (47.0-104.5) | 58 (42-75) | 48 (33.3-105.3) | 53.0 (41.0 - 92.5) | 74.5 (51.0 - 104.3) |
| Camps reported | 1 (1-1.3) | 2 (1-2) | 3 (2-3) | 1 (1-1) | 1 (1-2) | 2 (1-2) | 3 (2-3) | 1 (1-1) |
| Non-HH members present | 91.7 (22/24) | 85.0 (17/20) | 66.7 (2/3) | 100 (11/11) | 88 (22/25) | 89 (16/18) | 66.7 (2/3) | 100 (12/12) |
| People at camp (#) | 4-6 (4-6: 7-10) | 4-6 (4-6: 7-10) | 4-6 (1-3: 7-10) | 4-6 (1-3: 4-6) | 4-6 (4-6:7-10) | 4-6 (4-6:7-10) | 4-6 (1-3, 7-10) | 4-6 (1-3:4-6) |
| Nearby water source^*^ |  |  |  |  |  |  |  |  |
| Open^1^ | 83.3 (20/24) | 85.0 (17/20) | 100 (3/3) | 90.9 (10/11) | 80 (20/25) | 83.3 (15/18) | 100 (3/3) | 91.7 (11/12) |
| Closed^2^ | 42.7 (10/24) | 20 (4/20) | 33.3 (1/3) | 36.4 (4/11) | 36 (9/25) | 22.2 (4/18) | 33.3 (1.3) | 41.7 (5/12) |
| Animals traveled with |  |  |  |  |  |  |  |  |
| Goats | 100 (24/24) | 95 (19/20) | 100 (3/3) | 100 (11/11) | 100 (25/25) | 94.4 (17/18) | 100 (3/3) | 100 (12/12) |
| Sheep | 87.5 (21/24) | 80 (16/20) | 100 (3/3) | 100 (11/11) | 84 (21/25) | 83.3 (15/18) | 100 (3/3) | 100 (12/12) |
| Camels | 12.5 (3/24) | 5 (1/20) | 0 (0/3) | 9.1 (1/11) | 12 (3/25) | 5.6 (1/18) | 0 (0/3) | 8.3 (1/12) |
| **GPS details** |  |  |  |  |  |  |  |  |
| Campsite changes | 4 (2 - 8.3) | 17 (11-32.8) | 0 (0-3.5) | 0 (0-1) | 6 (2-9) | 17 (9.3-29.3) | 0 (0-3.5) | 0 (0-1.3) |
| Campsites logged | 3 (3-5.3) | 10.5 (7.3-18) | 1 (1-3) | 1 (1-2) | 4 (3-6) | 10.5 (5.0-18.5) | 1 (1-3) | 1 (1-2.3) |
| Total distance between camps (km) | 29 (11- 53) | 88 (70-210) | 2 (1-34) | 2 (2-6) | 37 (15-55) | 88 (72-206) | 2 (1-34) | 2 (1-5) |
| Total distance traveled (km) | 107 (36-157) | 279 (186-557) | 157 (131-186) | 34(23-55) | 114 (70-166) | 239 (184-536) | 150 (118-181) | 17 (11-48) |
