# Supplementary material for: Characterizing mobility patterns and malaria risk factors in semi-nomadic populations of Northern Kenya
Source: PLOS Glob Public Health. 2024 Mar 13;4(3):e0002750. doi: 10.1371/journal.pgph.0002750 (PMC10936864; doi:10.1371/journal.pgph.0002750)
Supplement: S2 Table — (DOCX) [file pgph.0002750.s005.docx]

**S2 Table.** Characteristics of Long Term and Transient trip types across three definitions of long term camps.

|  | **2 consecutive nights** | | **7 consecutive nights** | | **14 consecutive nights** | |
| --- | --- | --- | --- | --- | --- | --- |
|  | **Long term:**  **(n = 40)** | **Transient:**  **(n=4)** | **Long term:**  **(n=24)** | **Transient:**  **(n=20)** | **Long term:**  **(n=13)** | **Transient:**  **(n=31)** |
| **Traveler details** |  |  |  |  |  |  |
| Male | 92.5 (37/40) | 100 (4/4) | 87.5 (21/24) | 100 (20/20) | 84.6 (11/13) | 96.8 (30/31) |
| Age (years) | 37 (30-46) | 39 (37 - 41) | 37 (30-49) | 36 (29 - 36) | 40 (30-50) | 36 (30-42) |
| Malaria at Follow-up | 11.1 (4/36) | 0 (0/4) | 13.6 (3/22) | 5.6 (1/18) | 9.1 (1/11) | 10.3 (3/29) |
| **Catchment area** |  |  |  |  |  |  |
| Kangirisae | 30 (12/40) | 25 (1/4) | 29.2 (7/24) | 30 (6/20) | 30.8 (4/13) | 29.0 (9/31) |
| Lowae | 22.5 (9/40) | 25 (1/4) | 37.5 (9/24) | 5 (1/20 | 38.5 (5/13) | 16.1 (5/31) |
| Nakurio | 17.5 (7/40) | 50 (2/4) | 8.3 (2/24) | 35 (7/20) | 7.7 (1/13) | 25.8 (8/31) |
| Kerio | 30 (12/40) | 0 (0/4) | 25 (6/24) | 30 (6/20) | 23.1 (3/13) | 29.0 (9/31) |
| **Trip details** |  |  |  |  |  |  |
| Trip duration (days) | 54 (30.2-75.5) | 94.5 (61.8-123.0) | 56 (41.5-77.5) | 55 (33.8 - 83.8) | 59 (49-77) | 54 (33.5 - 76.0) |
| Camps reported | 1 (1-2) | 1.5 (1-2) | 1 (1-1.3) | 2 (1-2) | 1 (1-1) | 1 (1-2) |
| Non-HH members present | 87.5 (35/40) | 100 (4/4) | 91.7 (22/24) | 85.0 (17/20) | 84.6 (11/13) | 90.3 (28/31) |
| People at camp (#) | 4-6 (4-6:7-10) | 4-6 (1-3:7-10) | 4-6 (4-6:7-10) | 4-6 (4-6:7-10) | 4-10 (1-3:4-10) | 4-6 (4-6:7-10) |
| **Nearby water source** |  |  |  |  |  |  |
| Open | 82.5 (33/40) | 75 (3/4) | 83.3 (20/24) | 85.0 (17/20) | 72.7 (11/13) | 80.6 (25/31) |
| Closed | 30 (12/40) | 50 (2/4) | 42.7 (10/24) | 20 (4/20) | 38.5 (5/13) | 29.0 (9/31) |
| **Animals traveled with** |  |  |  |  |  |  |
| Goats | 97.5 (39/40) | 100 (4/4) | 100 (24/24) | 95 (19/20) | 100 (13/13) | 96.8 (30/31) |
| Sheep | 85 (34/40) | 75 (3/4) | 87.5 (21/24) | 80 (16/20) | 84.6 (11/13) | 83.9 (26/31) |
| Camels | 10 (4/40) | 0 (0/4) | 12.5 (3/24) | 5 (1/20) | 23.1 (3/13) | 3 (1/31) |
| **GPS details** |  |  |  |  |  |  |
| Campsite changes | 9 (3-12.8) | 51.5 (38-59.3) | 4 (2-8.3) | 17 (11-32.8) | 2 (2-7) | 12 (8.5-23.5) |
| Campsites logged | 5 (3-9) | 24 (20.3-26.8) | 3 (3-5.3) | 10.5 (7.3-18) | 3 (2-4) | 8 (5-14) |
| Total distance between camps (km) | 53 (24-80) | 276 (245-335) | 29 (11-53) | 88 (70-210) | 26 (8-53) | 74(41-177) |
| Total distance traveled (km) | 151 (75-229) | 693 (523-878) | 107 (36-157) | 279 (186-557) | 104(29-120) | 201.4 (140-496) |
