# Supplementary figures and images for: Characterizing mobility patterns and malaria risk factors in semi-nomadic populations of Northern Kenya
Source: PLOS Glob Public Health. 2024 Mar 13;4(3):e0002750. doi: 10.1371/journal.pgph.0002750 (PMC10936864; doi:10.1371/journal.pgph.0002750)

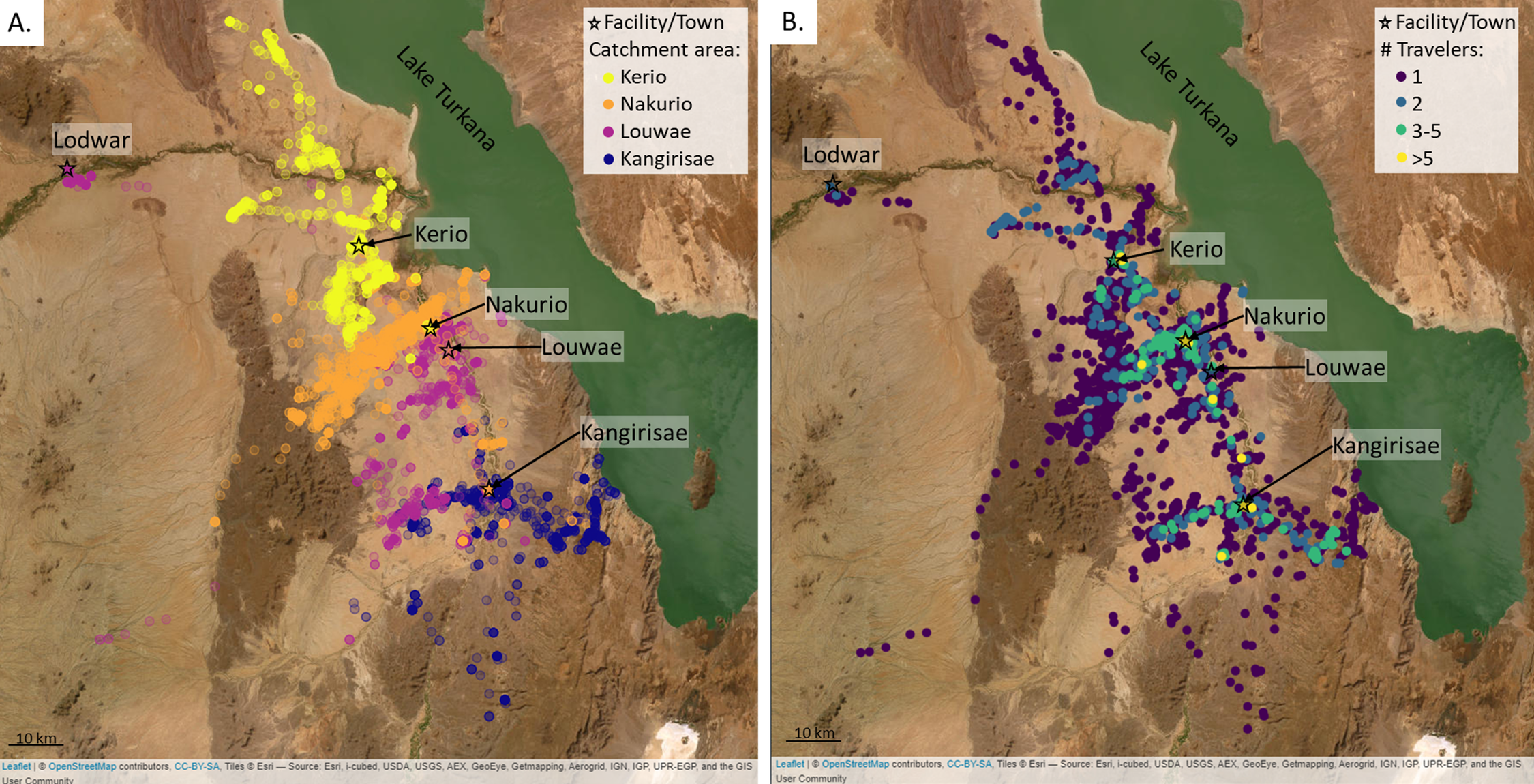

Supplement: S1 Fig — (A) All locations logged, stratified by traveler’s catchment area shows regionality. (B) Campsite locations, colored by the number of households logged at a given location to show areas commonly visited. Satellite image from Leaflet package in R, sourced by Esri. (TIF) [file pgph.0002750.s006.tif]

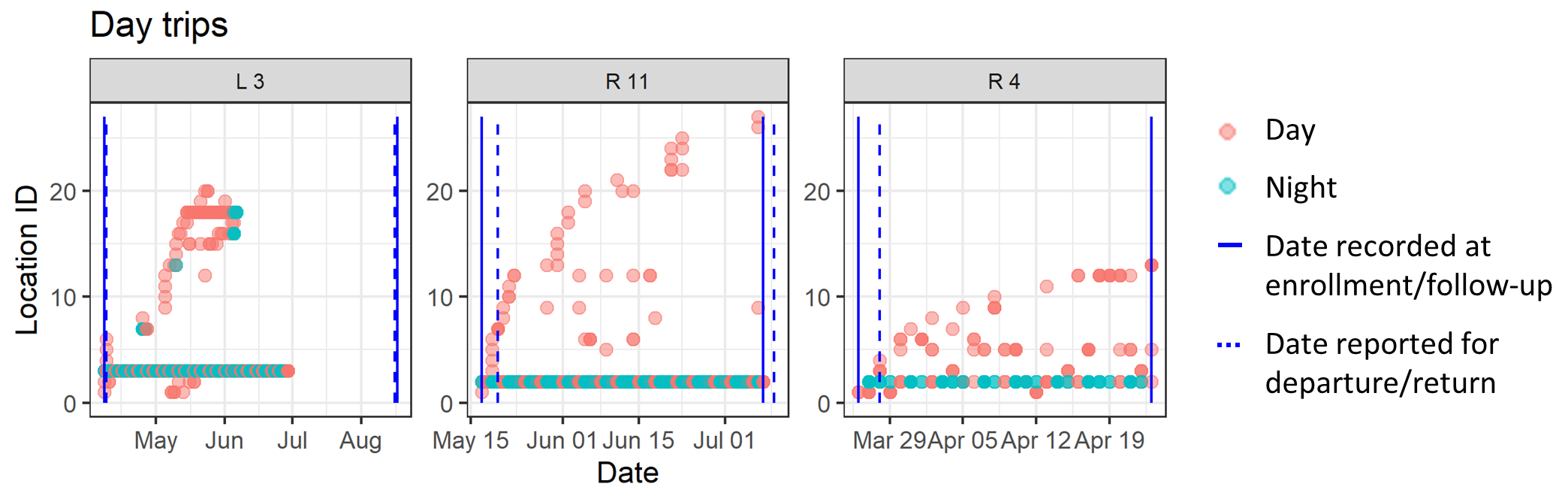

Supplement: S2 Fig — >90% of the night spots were spent at the same night location they were enrolled at, but most day points were logged at different locations. (TIF) [file pgph.0002750.s007.tif]

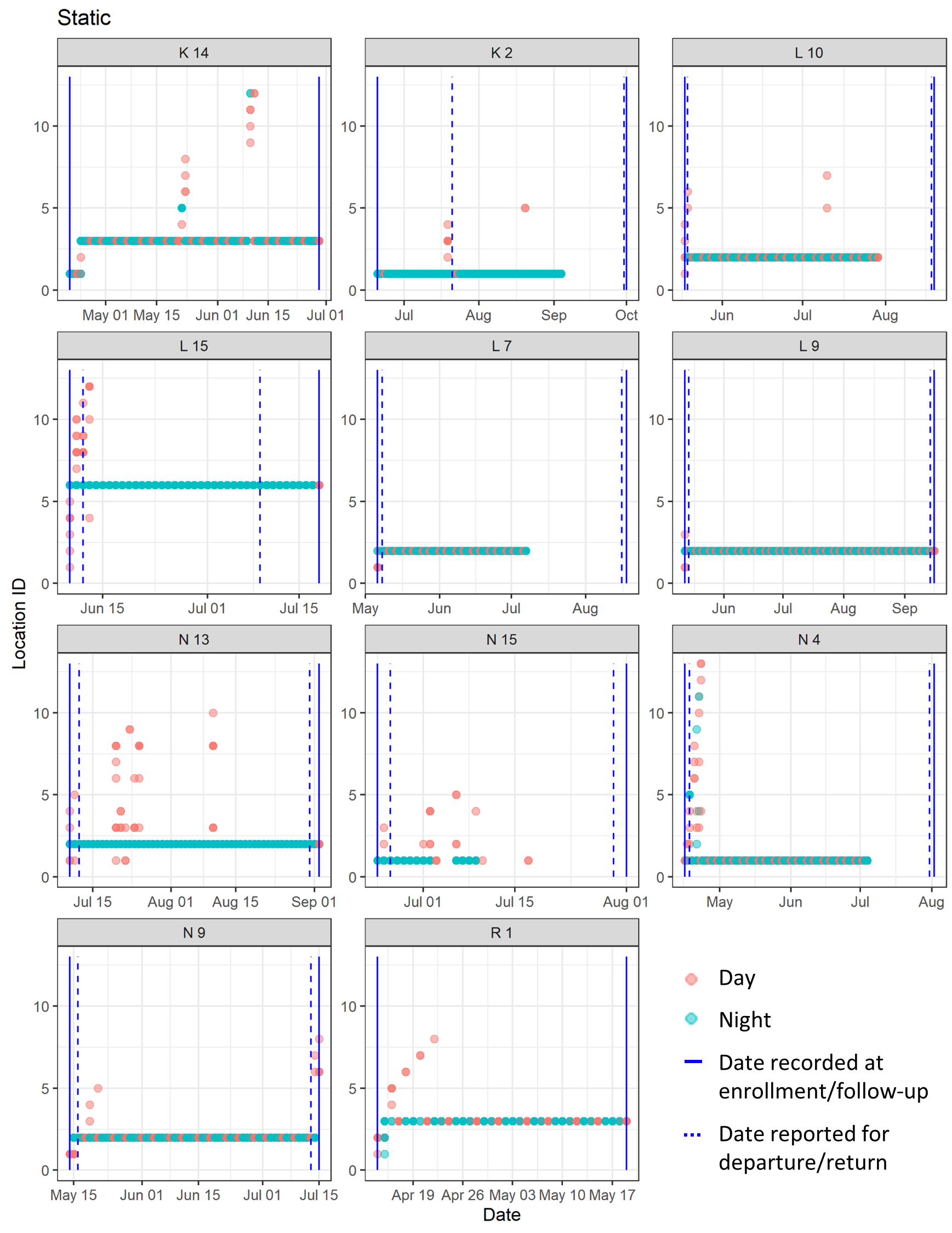

Supplement: S3 Fig — >90% of the night spots were spent at the same location and most night and day points were logged at the same location. (TIF) [file pgph.0002750.s008.tif]

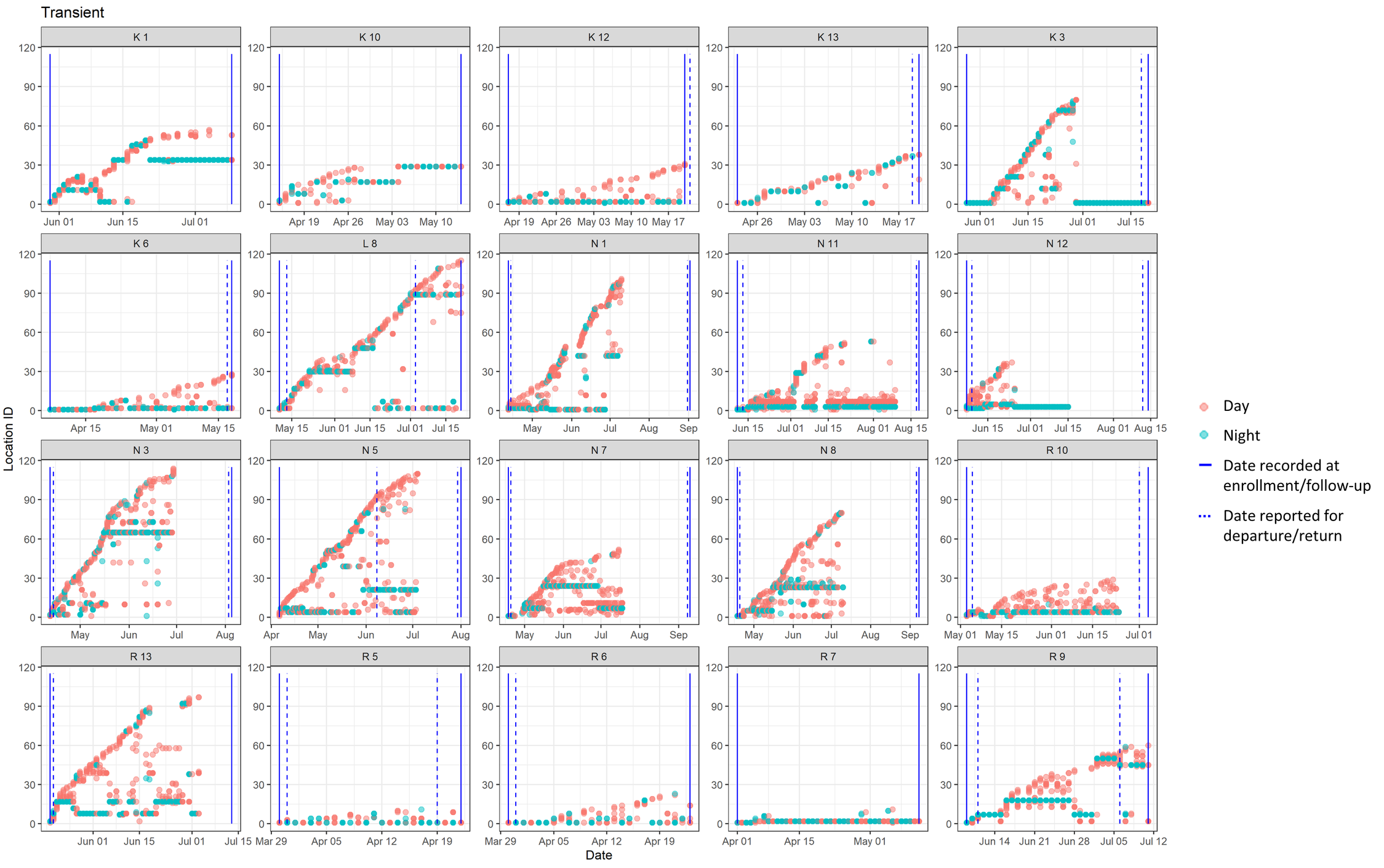

Supplement: S4 Fig — <90% of the night spots were spent at the same location and > 50% of nights were spent at transient camps (defined as camps with < 7 consecutive nights spent). (TIF) [file pgph.0002750.s009.tif]

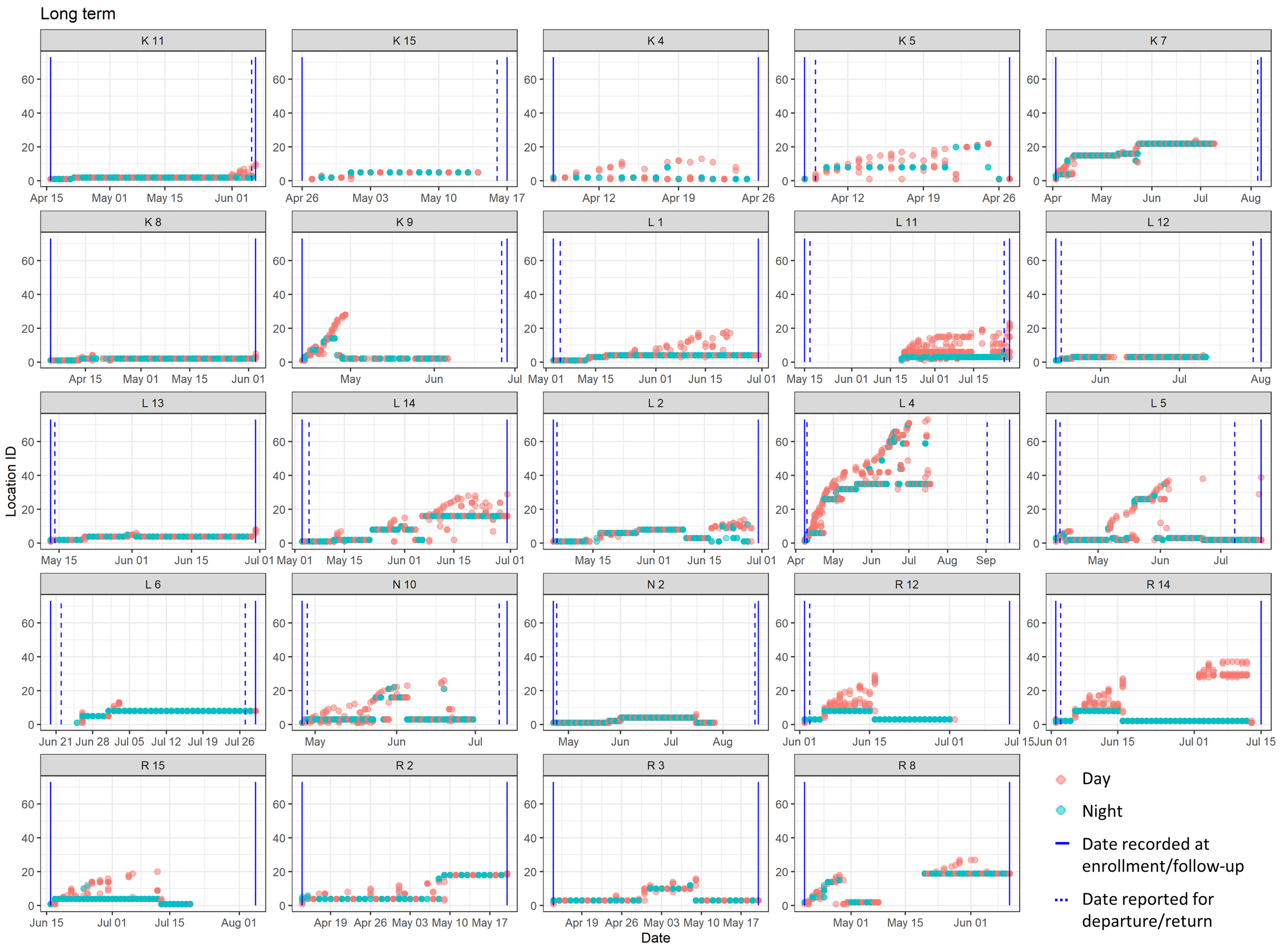

Supplement: S5 Fig — <90% of the night spots were spent at the same location and > 50% of nights were spent at long term camps (defined as camps with >7 consecutive nights spent). (TIF) [file pgph.0002750.s010.tif]

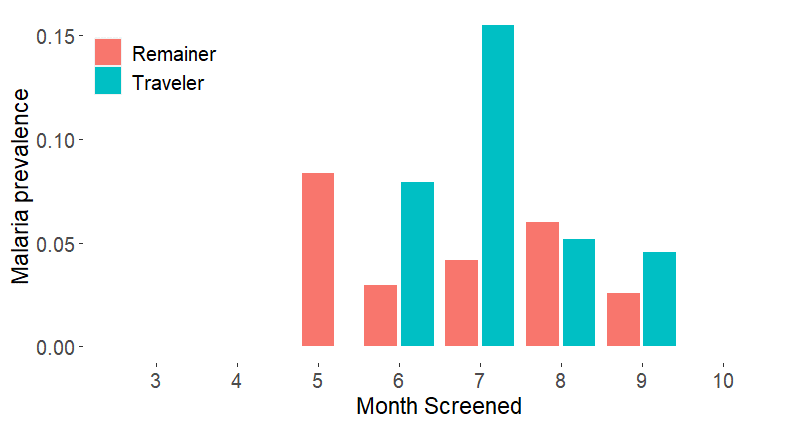

Supplement: S6 Fig — (TIF) [file pgph.0002750.s011.tif]
