# Supplementary material for: Characterizing mobility patterns and malaria risk factors in semi-nomadic populations of Northern Kenya
Source: PLOS Glob Public Health. 2024 Mar 13;4(3):e0002750. doi: 10.1371/journal.pgph.0002750 (PMC10936864; doi:10.1371/journal.pgph.0002750)
Supplement: Attachment — Submitted filename: Turkana nomads and malaria_response to PGPH reviewers.docx [file pgph.0002750.s013.docx]

Dear Dr. Abhinav Sinha,	February 8, 2024

We wish to submit our revised manuscript, “Characterizing mobility patterns and malaria risk factors in semi-nomadic populations of Northern Kenya” (PGPH-D-23-02422), by Meredith *et al*. We appreciate the reviewer’s feedback. Please see our point-by-point response below and the revised manuscript, with revisions tracked.

Please do let us know if you have any more comments.

Kind regards,

Hannah Meredith

Journal Requirements:

1. Please include a complete copy of PLOS’ questionnaire on inclusivity in global research in your revised manuscript. Our policy for research in this area aims to improve transparency in the reporting of research performed outside of researchers’ own country or community. The policy applies to researchers who have travelled to a different country to conduct research, research with Indigenous populations or their lands, and research on cultural artefacts. The questionnaire can also be requested at the journal’s discretion for any other submissions, even if these conditions are not met.  Please find more information on the policy and a link to download a blank copy of the questionnaire here: <https://journals.plos.org/globalpublichealth/s/best-practices-in-research-reporting>. Please upload a completed version of your questionnaire as Supporting Information when you resubmit your manuscript.

We have uploaded a completed version of the questionnaire.

Reviewers' comments:

Reviewer's Responses to Questions

**Comments to the Author**

1. Does this manuscript meet PLOS Global Public Health’s [publication criteria](https://journals.plos.org/globalpublichealth/s/criteria-for-publication)? Is the manuscript technically sound, and do the data support the conclusions? The manuscript must describe methodologically and ethically rigorous research with conclusions that are appropriately drawn based on the data presented.

Reviewer #1: Partly

Reviewer #2: Yes

2. Has the statistical analysis been performed appropriately and rigorously?

Reviewer #1: No

Reviewer #2: Yes

3. Have the authors made all data underlying the findings in their manuscript fully available (please refer to the Data Availability Statement at the start of the manuscript PDF file)?

The [PLOS Data policy](https://journals.plos.org/globalpublichealth/s/data-availability) requires authors to make all data underlying the findings described in their manuscript fully available without restriction, with rare exception. The data should be provided as part of the manuscript or its supporting information, or deposited to a public repository. For example, in addition to summary statistics, the data points behind means, medians and variance measures should be available. If there are restrictions on publicly sharing data—e.g. participant privacy or use of data from a third party—those must be specified.

Reviewer #1: No

Reviewer #2: Yes

We will have submitted the dataset and code needed to reproduce most of the tables and figures in this manuscript to the Duke repository. To protect the privacy of the participants in this study, we have removed the GPS logger coordinates. We will update the manuscript to include the DOI as soon as it is available.

4. Is the manuscript presented in an intelligible fashion and written in standard English?

PLOS Global Public Health does not copyedit accepted manuscripts, so the language in submitted articles must be clear, correct, and unambiguous. Any typographical or grammatical errors should be corrected at revision, so please note any specific errors here.

Reviewer #1: Yes

Reviewer #2: Yes

5. Review Comments to the Author

Please use the space provided to explain your answers to the questions above. You may also include additional comments for the author, including concerns about dual publication, research ethics, or publication ethics. (Please upload your review as an attachment if it exceeds 20,000 characters)

Reviewer #1: The paper aims to evaluate mobility patterns and their role in determining the risk of malaria exposure in semi-nomadic populations in Central Turkana, Kenya. The theme is intriguing, and further investigation is warranted in this area, reinforcing the justification for developing this study. The structure is well-defined, encompassing introduction, study area, data, study design, results, discussion, and conclusion. However, I have some concerns regarding the content:

ABSTRACT

- The abstract could emphasize the malaria burden in the country, the study region, and within this mobile group, underscoring the significance of the study for this specific population.

Thank you for the suggestion. We have added a line in the abstract about malaria in this region.

STUDY DESIGN

- Consider addressing malaria seasonality in the study location. Why wasn't the survey conducted throughout the entire year? Does the study period capture the peak of yearly malaria transmission?

Based on prior work, malaria is found year-round in the settled communities in Central Turkana(1). Funding limited the number of households enrolled and duration of study, so we opted for a time window when we could enroll the greatest number of households that had at least one traveler intending to migrate with their herds (as opposed to a time of year when travelers are less likely to leave with their herds because conditions are favorable around their homestead). This allowed for the comparison of malaria prevalence between the travelers and remainers. It would be interesting to characterize seasonal malaria dynamics in semi-nomadic communities, but this would require at least two years of surveillance to identify seasonal trends and was outside the scope of this study. We have updated the text to include better justification around the study time window selected and the suggestion of characterizing seasonal trends in future studies.

- Information needed: Provide details on the conditions where these households are located. Are they more protected to malaria vectors and parasites than other areas these populations travel to?

It depends. Based on malaria detected at enrollment and anecdotes from community health workers, malaria appeared to impact some areas more than others (i.e. Nakurio community members frequently reported malaria cases in town and asked for bednets, whereas Kangirisae did not).

Generally, households are in dry, sandy areas and often under/near a grove of trees. Distance to water sources varies. Temporary structures made of palm fronds and sticks (bomas) were not always present, and, if they were, members tended to only use them for storage or shelter from the sun during the day. Households typically sleep outside at night and the ones enrolled reported no access to bednets.

We anticipate that travelers experienced similar conditions – traveling in dry, sandy areas, sleeping outside without bednets; however, the main difference was that they were potentially seeking out areas to feed and water their herds (i.e. areas that had recent rainfall or a borehole). Rainfall in Turkana is often short and intense and results in rapid growth of shrubs and accumulation of water that travelers seek out for their herds. It is likely that these conditions could have attracted mosquitoes/facilitated breeding sites that would then be more conducive to malaria transmission. Further work is needed to characterize the conditions of different locations visited and compare them to the homestead conditions.

More details around these conditions have been added to the methods (“Study Area”) to provide readers with more context.

- Information needed: Discuss the vector in this area and main breeding sites. Detail vector habits (night, day, sunset, dusk, all day) and explore how the vector is related to mobile populations. Are entomologic data available? If not how can this challenge be circumvented?

There is a gap in knowledge around the vectors and their habitats across Turkana. Until recently, Turkana was assumed to have little malaria. Consequently, malaria vectors in this county have not been well documented. In 2010, Okara et al surveyed the distribution of malaria vectors in Kenya(2). Only two surveillance sites were in Turkana county’s 100,000 km^2^ (neither of which were close to this study’s site) and they were only surveying for *Anopheles arabiensis*, the vector associated with the malaria outbreaks in the Kakuma refugee camp(3).

Work is being done to start filling the knowledge gap around malaria vectors in Turkana. A recent study by Ochomo et al indicates that *Anopheles stephensi* are present in Lodwar, the capital(4). However, as *A.stephensi* are predominantly found in urban settings, we cannot be certain they were also present in the more rural areas where the participants were located.

Given the nascent stage of knowledge around vectors in this region, this study was focused more on determining if traveling with herds was a risk factor for acquiring malaria, regardless of vector. To recommend suitable interventions, an important next step would be to better characterize the vectors and their habitats across Turkana and determine which ones are most likely encountered by semi-nomadic communities. The Discussion has been updated to describe the need to better characterize vectors and how that could impact intervention strategies.

- In connection with the preceding point, let's revisit the hypothesis: “Since nomadic individuals traveling with their herds tend to congregate at water sources, potential mosquito breeding sites, we hypothesized that the Turkana who travel with their herds are more likely to be exposed to malaria than those who remained in the settled communities.” Has this hypothesis been rigorously tested? Considering the utilized data, it appears that the results might not adequately address this hypothesis.

The reviewer is correct – we do not have sufficient information to conclude that more travelers had malaria because they congregated at a water source that was likely a breeding source for mosquitoes. However, we did collect sufficient data to address the hypothesis that a Turkana who travels with their herd will be more likely to have malaria after the migration period than a Turkana who remains behind. Our rationale behind this hypothesis was based on the understanding that 1) Turkana migrate in search of food and water for their herds and 2) water sources are a known breeding site for mosquitoes that could be involved in transmitting malaria. However, because we do not have the evidence that the water sources visited during migration were the source of mosquitoes that infected the travelers**,** we will update the text by unlinking our initial rationale from the hypothesis.

- Explain the process of household selection and justify why these specific catchment areas were chosen. Provide information on the total population in the selected catchment areas and discuss the sample's representativeness concerning location.

These communities were chosen because of the high proportion of households who travel with their herds compared to communities closer to the Turkwel river. Ministry of Health colleagues and community health workers helped us identify catchment areas with an abundance of semi-nomadic households. Regarding the household selection process, community health workers from a given catchment area would get word that a household was about to leave with their herd. CHWs would screen the household to determine eligibility for enrollment. We have updated the Methods to reflect these points.

The total population in these catchment areas fluctuates seasonally, but the approximate number of households was 3800. We have added this to the description of the study area. From our previous work, we determined that 93.4% (191/204) of households are semi-nomadic in this area (unpublished). Although our sample may be biased towards those who travel earlier in the season or are better known by the CHW, it is unlikely that this bias is related to the outcome but may affect generalizability. We have discussed the potential bias in the limitations.

- Discuss why community health workers were responsible for participant identification and enrollment. Were they adequately trained? Why not have research scientists conduct these activities to minimize bias/errors?

Community health workers were appointed because they are trusted community members of a vulnerable and marginalized population, they speak the local language, and they are in communication with the households to know when they will come and go on their migrations. They were in a unique position to help us identify participants for this research. Working through research scientists would have presented a power differential, and would not have had the local network to quickly know when a traveler was getting ready to leave on migration.

Community health workers were selected and overseen by heads of each Health Facility. Each CHW and Health Facility head (total of 12) participated in an intensive 2-day workshop where they were trained in the study’s purpose, household and participant eligibility criteria, informed consent, how to perform finger pricks and create dried blood spots. Furthermore, we met with the CHWs regularly to refresh their skills and review every data collection tool in order to minimize errors and missing data. We found no evidence of data manipulation and although we did find errors and omissions from time to time, they adhered well to the inclusion criteria.

- Concern: At enrollment, before the travelers left with their herds, and again at follow-up, after the travelers returned with their herds, participants provided a finger-prick blood sample for a dried blood spot (DBS) and answered a questionnaire detailing their travel and medical history”. Depending on the duration of their absence, malaria parasites might become undetectable upon their return. In the event of infection during travel, symptoms or parasitemia could become indiscernible. How was this challenge addressed, and what impact did it have on the results?

As part of the follow-up survey, we asked participants to detail their health since they were enrolled. We asked questions such as:

- “Have you felt sick since enrollment?”,
- “What did you do if you felt sick?”
- “Were you tested for malaria?”
- “Did you take any medication?”

Very few participants reported feeling ill (16/671 or 2.4%) since enrollment. Only 4 participants reported testing positive during the study, all of whom had positive PCR results at follow-up despite reporting taking an antimalarial. This is consistent with previous findings that parasite DNA can be detected up to 42-48 days after successful treatment(5).

If left untreated, Plasmodium infections can last multiple years, in which case PCR should be able to detect it for the timeline of this study (longest trip length reported was 188 days)(6)

RESULTS

- Table 1. As an illustration, consider the comparison between static and transient groups. The former, characterized by no campsite changes, a smaller distance between camps, and lower overall travel distance, may face an increased risk of malaria infection if they remain static in an area where they are more exposed to the malaria vector. In such cases, their likelihood of being infected with malaria parasites would rise. On the contrary, the transient group has the flexibility to move to other areas with a lower likelihood of infection, thereby protecting themselves from getting infected.
To gain a comprehensive understanding, it is essential to determine the endemicity of the areas where these populations move and associate it with their mobility patterns. Additionally, this information should be compared with the endemicity of the locations where the remainers, who have not moved, are situated. Without such considerations, we risk making assumptions about how mobility influences malaria infection rates, potentially overlooking key conditioning factors that may not have been measured.

We agree with the reviewer’s point. As noted in the introduction, mobility can bring people into or away from high transmission areas. We assumed the prevalence of malaria in remainers served as a proxy for the endemicity in those catchment areas and compared these prevalences in Table 3 and text:

*“The catchment area with the lowest number of infections was Louwae for both groups (7.1% (4/56) of travelers and 3.1% (4/127) of remainers) while the catchment areas with the highest was Nakurio for travelers (11.1% (6/54)) and Kerio for remainers (5.8% (7/120)). There was one month where malaria was more common in remainers followed up in that month than in returning travelers; otherwise, malaria infection in travelers was similar to or greater than the prevalence in remainers in all the study months.“*

Unfortunately, collecting data on the endemicity of the areas visited by the travelers was outside the scope of this study; however, the study showed that the travelers had higher prevalence of malaria after their trips than the remainers across each catchment area. While we do note that we still do not fully understand where the travelers were acquiring malaria, we will further emphasize the need to characterize malaria across Turkana to better understand how traveling to specific areas could impact malaria infection rates.

- Table 2. Have other vector control strategies been implemented, such as indoor residual spraying? Is malaria treatment readily available, and if so, how accessible is it? In terms of water sources, are there locations where both open and closed water sources are nearby, or is it a situation where only one type is available and not the other?

Since Turkana has been assumed to not support malaria transmission until very recently, very few vector control strategies have been allocated for this region either because malaria transmission has historically been assumed negligible or the interventions are not suitable for semi-nomadic communities. For example, indoor residual spraying is often not relevant in these communities as households tend to sleep outdoors. The structures they build to store supplies and seek shade under are made from sticks and palm fronds and are not solid, so spray would not be very effective. As such, IRS and other vector controls have not been implemented in Turkana.

Malaria treatment is available at health facilities; however, health facilities are not easily accessible to travelers when they are migrating with their herds. Furthermore, many of the cases detected in this area are asymptomatic and/or have parasitemia levels below the detection limit of the rapid diagnostic tests used.

There are locations where both open and closed water sources are available. Tables 1-3 report the number of households/individuals who reported use of any “Open” or “Closed” sources (i.e. households could report one or both). The footnote of Table 1 clarifies this.

- Table 3. We aim to clearly understand the prevalence of malaria among travelers during both the baseline and follow-up surveys. Additionally, we seek to determine the malaria prevalence among remainers at both baseline and follow-up.

This study focused on understanding the malaria acquired during the period of migration. As such, the text and Table 3 considered participants who were malaria negative at baseline. Participants with malaria at enrollment were excluded from analysis, as detailed in Figure 2 and the text.

- Table 3. To assess whether there is a statistically significant difference in characteristics between travelers and those who remained, a chi-square test could be employed to compare PCR+ results. This test would help determine if the observed percentages are statistically significant, providing insight into the distinctions between individuals who acquired malaria while traveling versus those who stayed in one location.

To investigate statistically significant factors associated with malaria, we ran univariate and multi-variate models (Table 4). The univariate model suggests that the difference in malaria prevalence due to travel status was statistically significant (OR 2.19 with 95% confidence interval of 1.15-4.18), just as a chi-square test does (p = 0.0235). Chi-square tests cannot be adjusted for other variables, which is why we decided to use multi-variate models instead. We have not included the chi-square test because it would be redundant with what is currently reported.

- Table 3. Clarify the term "sick since enrollment" in Table 3 and explain the criteria for sickness.

Please see answer to next comment.

- Table 3. What does "sick since enrollment" entail? Does it imply that individuals retained symptoms from the enrollment survey until the follow-up survey? This interpretation appears puzzling. Perhaps the intended expression is "sick at enrollment." Furthermore, the term "sick" requires clarification – does it refer to being diagnosed with malaria, and is this diagnosis based on PCR conducted by the research project?

Sick since enrollment is asking whether they had any new illness episode between enrollment and followup. Because semi-nomadic household members may not 1) have access to or 2) chose to seek out a medical diagnosis and treatment from a health facility, we asked if they had felt sick in the time since they were enrolled to gain insight on their health between screenings at enrollment and follow-up. If they reported feeling sick during the time between enrollment and follow-up, we asked additional questions to try to understand if it might have been malaria or something else and if they had received treatment for their malaise (Table 3). We separately asked about symptoms on the day of enrollment and follow-up (reflected by the line in Table 3 “No symptoms on day screened”). We have updated the text to clarify what “sick since enrollment” means.

- Table 4. Discuss the lack of statistically significant results in the multivariate model. Consider exploring other variables not used in the modeling (trip types, catchment area, water source, income source, etc.).

The lack of statistically significant results from the multi-variable model is largely due to sample size. The model presented included three factors: travel status because that was the variable we were interested in determining a relationship with malaria for; age because it is a known risk factor for malaria; and gender, because it was a significant factor in univariate analysis. Other factors had been analyzed, but were not included because they were statistically insignificant in univariate analysis. Trip types were not included, as further explained in the next answer. The text has been updated to better explain the methods/rationale behind this analysis.

|  | **OR (95% CI)** | **aOR (95% CI)** |
| --- | --- | --- |
| **Travel Status** |  |  |
| Remainer | ref | ref |
| Traveler | 2.19 (1.15-4.18)* | 1.91 (0.86 - 4.32) |
| **Age (years)** |  |  |
| ≤15 | ref | ref |
| 16-40 | 0.81 (0.39 - 1.69) | 0.69 (0.32 - 1.52) |
| >40 | 1.14 (0.44 - 2.76) | 0.86 (0.31 - 2.22) |
| **Gender** |  |  |
| Female | ref | ref |
| Male | 2.16 (1.11 - 4.40)* | 1.54 (0.70 - 3.48) |
| **Catchment** |  |  |
| Kangirisae | Ref |  |
| Kerio | 1.07 (0.43 – 2.69) |  |
| Louwae | 0.67 (0.24-1.79) |  |
| Nakurio | 0.99 (0.40 – 2.54) |  |
| **Water** |  |  |
| Closed only | Ref |  |
| Open only | 1.77 (0.87-3.91) |  |
| Open and closed | 0.73 (0.16-2.46) |  |

- Table 4. The analysis excluded trip types due to small group sizes. Ideally, a more effective approach would involve consolidating trip types into two groups, each with a higher population. The isolated inclusion of day trips, with only three observations, may not provide meaningful insights on its own, potentially hindering the identification of patterns. If the number of observations per trip type is insufficient, the study's design could benefit from an enhanced enrollment process. Alternatively, reconsidering the feasibility of conducting statistical modeling analyses might be warranted.

Only 44/218 travelers carried a GPS logger and were included in the malaria analysis. Of these, 4 tested positive for malaria at follow-up. Despite the low case count, we were able to identify a pattern: all four cases were in travelers who spent > 50% of their nights away from the homestead (1 transient and 3 long term trips). As the reviewer rightly points out, this analysis would benefit from an increased sample size; however, funding limited the number of GPS loggers the study could afford which in turn limited the number of GPS tracks that could be recorded. The travel surveys could have helped categorize trip types without a GPS logger; however, as noted below, there were discrepancies between what was recorded by GPS logger and what was reported in the survey that prevented us from extrapolating trip types from the surveys.

*“To further characterize these trip patterns, travel history from the surveys was incorporated. For most trip types, travel surveys tended to underestimate the number of campsites calculated from the GPS loggers (Table 1) and did not distinguish between the distances covered (i.e., using travel time to a camp as a proxy for distance). These differences possibly reflect recall bias and suggest that a travel survey alone might not detect the nuances of different trip types.”*

For these reasons, trip type was left out of the model.

DISCUSSION/CONCLUSIONS

- Expand the limitations section to include lines 341-359.

Thank you for the suggestion. We will indicate that the recommended areas for additional research are inspired by the limitations of our study.

- Develop policy implications further, in line with the introduction's emphasis on tailoring surveillance and intervention strategies for unique populations.

In the first paragraph of the discussion, we discuss a number of strategies that have been used in mobile populations in the past and why they are not necessarily realistic in this setting. We recommend:

*“Instead, focusing on preventative measures, such as bednet distribution in more remote areas would be conducive for reducing malaria transmission in travelers who spend most of their nights at the same place (i.e., Day trip or Static trips) and also prevent onward transmission from infected travelers upon their return. For travelers who spend more nights away from their settlements (i.e., Long term or Transient travelers), there is a need for solutions that would easily integrate into their lifestyle, such as insecticide treated clothing* (7)*. Ultimately, maintaining an open dialogue with mobile communities about their needs and co-developing practical solutions is critical to ensure interventions are maximally useful.”*

We have added some additional ideas around CHW engagement and the use of bednets specifically designed for mobile populations.

- Enhance the references, providing more information on the relationship between malaria and population mobility, beyond Africa.

We will update the discussion to more broadly contextualize the findings of this study.

I commend the authors for their efforts in developing this paper, and I hope that these suggestions will assist them in augmenting their contribution to the scientific literature.

We thank the review for their time and constructive feedback.

**Reviewer #2**: The MS is well written the GPS technology was used to track the population. Authors have tried to show that infection is acquired by the travelers. some of the observations on the MS are as follows:
Using the GPS was a problem to the human to carry them however, some of the GPS should have been fixed on livestock that might be helpful in case they forgot to carry with them.

We thank the reviewer for the suggestion. That is a creative solution; however, we opted to ask the people to carry the loggers so we could directly relate their movement to their malaria outcomes.

In the Methods it is not clear that RDTs were used or not if not : Why RDTs were not used to check any parasitiemea to treat any positive case during collection of Filterpaper blot this would have reduced the transmission in case of positivity

We thank the reviewer for their question. If participants felt unwell at enrollment or follow-up, community health workers offered them an RDT and referred them for appropriate care at the nearest health facilities. These RDTs were used for care rather than assessing research outcomes. This decision was largely based off of prior work(1), where the majority of asymptomatic cases were not detected by RDTs.

lines 250-252 how was malaria tested in the individuals through PCR only "?. how can it be sure they have not acquire infection from start point have if they were not sampled 10 days after also?

PCR is the most sensitive test for malaria, detecting infections 2-3 days faster than blood smears and rapid diagnostic tests(8,9). We cannot rule out the possibility that infections may have been acquired around the time of enrollment that were too nascent to be detected by PCR. Since the participants would leave on their treks shortly after being enrolled, we could not retest them 10 days later to determine their malaria status. However, the fact remains that participants who traveled with their herds were more likely to be infected at the end of the travel period than the remainers. This suggests that there is some trait associated with the participants who traveled that increases their risk of malaria infection.

lines 316-318 why there was higher malaria in males remainers while gender positivity insignificant in traveler

This is a good question and one that we are not completely certain about. In the discussion, we surmise that different gender norms could be more prominent in daily life around the homestead than while out herding their animals.

lines 357-358 : it was mentioned that hotspot were identified using GPS how ? and secondly malaria is transmitted by infected mosquitoes whether and how infected mosquitoes were available to these travelers are the areas where they stay were infection was already occurring?

Thank you for the opportunity to better define some of the methods. By “hotspot”, we meant a popular destination that many travelers visited. This could be determined by the analysis presented in Figures 3B and S1, where we overlayed the GPS logger tracks and determined where multiple individual’s tracks overlapped. We have updated the methods to clarify this and provided a more concrete definition of a hotspot along with a reference to support the definition(10).

overall the study could show the trac of nomadic population and need for their screening whereas was not able to show the areas where probably infection was possibly coming.
the study excluded PV which is even though with complication of relapse it is obvious and make sense to exclude Pv.
there was a need to assess the malaria situation of the areas where these nomadic population was staying during their movements.
Paper needs cognizing it seems lengthy paper.

We thank you for your comments and will try to condense the length where appropriate.

References:

1.	Meredith HR, Wesolowski A, Menya D, Esimit D, Lokoel G, Kipkoech J, et al. Epidemiology of Plasmodium falciparum infections in a semi-arid rural African setting: Evidence of reactive case detection in Northwestern Kenya. Am J Trop Med Hyg [Internet]. 2021 Oct 6 [cited 2021 Oct 27];105(4):1076–84. Available from: https://www.ajtmh.org/view/journals/tpmd/105/4/article-p1076.xml

2.	Okara RM, Sinka ME, Minakawa N, Mbogo CM, Hay SI, Snow RW. Distribution of the main malaria vectors in Kenya. Malar J [Internet]. 2010 Mar 4 [cited 2024 Jan 21];9(1):1–11. Available from: https://malariajournal.biomedcentral.com/articles/10.1186/1475-2875-9-69

3.	Bayoh MN, Akhwale W, Ombok M, Sang D, Engoki SC, Koros D, et al. Malaria in Kakuma refugee camp, Turkana, Kenya: Facilitation of Anopheles arabiensis vector populations by installed water distribution and catchment systems. Malar J [Internet]. 2011 Jun 4 [cited 2024 Jan 21];10(1):1–11. Available from: https://malariajournal.biomedcentral.com/articles/10.1186/1475-2875-10-149

4.	Ochomo EO, Milanoi S, Abong’o B, Onyango B, Muchoki M, Omoke D, et al. Detection of Anopheles stephensi Mosquitoes by Molecular Surveillance, Kenya. Emerg Infect Dis [Internet]. 2023 [cited 2024 Jan 21];29(12):2498. Available from: /pmc/articles/PMC10683825/

5.	Vafa Homann M, Emami SN, Yman V, Stenström C, Sondén K, Ramström H, et al. Detection of Malaria Parasites After Treatment in Travelers: A 12-months Longitudinal Study and Statistical Modelling Analysis. EBioMedicine [Internet]. 2017 Nov 1 [cited 2024 Jan 28];25:66–72. Available from: http://www.thelancet.com/article/S235239641730395X/fulltext

6.	Ashley EA, White NJ. The duration of Plasmodium falciparum infections. 2014 [cited 2024 Jan 28]; Available from: http://www.malariajournal.com/content/13/1/500

7.	Crawshaw AF, Maung TM, Shafique M, Sint N, Nicholas S, Li MS, et al. Acceptability of insecticide-treated clothing for malaria prevention among migrant rubber tappers in Myanmar: a cluster-randomized non-inferiority crossover trial. Malar J [Internet]. 2017 Feb 28 [cited 2023 Sep 4];16(1):92. Available from: /pmc/articles/PMC5329906/

8.	Mpina M, Stabler TC, Schindler T, Raso J, Deal A, Pupu LA, et al. Diagnostic performance and comparison of ultrasensitive and conventional rapid diagnostic test, thick blood smear and quantitative PCR for detection of low-density Plasmodium falciparum infections during a controlled human malaria infection study in Equatorial Guinea. Malar J [Internet]. 2022 [cited 2024 Jan 28];21:99. Available from: https://doi.org/10.1186/s12936-022-04103-y

9.	Walk J, Schats R, C Langenberg MC, Reuling IJ, Teelen K, Roestenberg M, et al. Diagnosis and treatment based on quantitative PCR after controlled human malaria infection. Malar J. 2016;15:398.

10.	Lessler J, Azman AS, McKay HS, Moore SM. Perspective Piece What is a Hotspot Anyway? Am J Trop Med Hyg [Internet]. 2017 [cited 2024 Jan 21];96(6):1270–3. Available from: http://www.ncbi.nlm.nih.gov/pubmed/5880862.
